# Editorial: Driving Innovation in Organic Optoelectronic Materials With Physics-Based and Machine-Learning *De Novo* Methods

**DOI:** 10.3389/fchem.2022.973254

**Published:** 2022-08-03

**Authors:** Paul Winget, Mathew D. Halls, Rafael Gómez-Bombarelli, Chihaya Adachi

**Affiliations:** ^1^ Schrödinger, Inc., New York, NY, United States; ^2^ Consultant at Kyulux NA, Massachusetts Institute of Technology, Cambridge, MA, United States; ^3^ Kyushu University, Fukuoka, Japan

**Keywords:** organic optoelectronics, OLED (organic light emitting diodes), OPV, TADF, machine learning (ML), artificial intelligence (AI), quantum chemistry, molecular dynamics

Organic materials are a rapidly evolving class of compounds for applications in current and future optoelectronic devices. Contributing to the swift pace of discovery of new materials are computational methods based on physics, machine learning, or an astute combination of both. This is a highly interdisciplinary research area where diverse approaches yield an assortment of different chemical motifs with the desired multi property design profile. Many of these chemistries would not have been identified using traditional approaches, often accompanied with new insights into fundamental structure-property relationships influencing device operation. This Research Topic brings together nine articles on the latest advances in this research area.

First, an article from (Desmedt et al.) takes up the challenge to design efficient molecular nonlinear optical (NLO) switches by means of an inverse design algorithm. Optimal functionalizations for both redox and topological hexaphyrin-based switches and reveals that (centro)symmetry emerges as the dominating factor driving the NLO response, together with the macrocyclic torsional strain. Introduction of differing numbers, types, and position of meso-substituents, leads to NLO contrasts up to 15 times larger for the redox-based switch and two times larger for the topological switch.

Then, the article by (Kaiser et al.) focuses on calculation of the carrier mobility in organic semiconductors, e.g., those in organic light-emitting diodes (OLEDs), organic photovoltaics (OPVs) and organic field-effect transistors (OFETs). These materials are derived from a vast chemical space which is impossible to explore using experiment alone. A dramatic complication is that these properties require knowledge of both molecular and morphological structure. This multiscale virtual screening approach allows a significant improvement in device performance prediction.

Efficiencies in OLEDs and OPVs was also of interest to (Yakubovich et al.). They developed a workflow to search for molecules suitable for the fusion of triplet-triplet excitations (triplet-triplet fusion, TTF) in blue OLED devices. Their work presents a comprehensive computational evaluation of several generative models. This approach yields a large fraction of generated molecules relevant for the problem of interest, thus greatly reducing the search space. The excitation energies were then predicted using a modification of the Junction Tree VAE (JT-VAE) neural network. The presented workflow is not limited to TTF materials and can be adopted for the discovery of other molecules for optoelectronic applications.

Lin et al. also investigate organic semiconductors in the context of thermally-activated delayed fluorescence (TADF). TADF is a concept which helps to harvest triplet excitations, boosting the efficiency of an organic light-emitting diode that can be observed in molecules with spatially separated donor and acceptor groups with a reduced triplet-singlet energy level splitting. For single layer TADF architectures to achieve high efficiency, materials with balanced electron and hole transport are required in addition to emission wavelength and efficiency. Interestingly, differing excitation characteristics in the singlet and triplet excited states were included in the virtual screen.

Kwak et al. demonstrated the use of recurrent neural network-based generative machine learning techniques combined with high-throughput quantum chemical simulations. An exhaustive structural enumeration was followed by generating new machine-generated design ideas in a shifted chemical property space. That property space is generated by a utility function which combines multiple optoelectronic parameters design directives into a multiparameter optimization (MPO) score. A notable quality is the dramatic improvement in the MPO score without a corresponding increase in similarity to the training set. This demonstrates the ability to generate new design ideas rather than interpolate from and optimize previous work.

An alternative to exhaustive physics-only screening was presented by (Abroshan et al.). Building reliable predictive ML models requires creating and managing a high volume of data that adequately address the complexity of materials’ chemical space. Active learning (AL) was presented as a strategy to efficiently navigate the search space by prioritizing the decision-making process for unexplored data. This approach allows a more systematic mechanism to identify promising candidates by minimizing the number of computations required to explore an extensive materials library with diverse variables and parameters. An example identifying materials candidates for hole-transport layers (HTL) in OLEDs utilized this AL workflow.

While many of the articles focus on the optoelectronic metrics (Hauenstein et al.), focuses on the lifetime of the resulting device. Three-dimensional kinetic Monte Carlo (KMC) simulations are compared to an experimentally well-characterized efficient green-emitting device. A comparison of the simulated and experimental time-dependence of the luminance decay provides the probability that a degradation-triggering event, e.g., exciton-polaron quenching and exciton-exciton annihilation processes, leads to the formation of a degraded molecule. This approach enables systematic studies of the operational lifetime and its sensitivity to the material composition, layer structure, and charge carrier balance.

Kinetic Monte Carlo (KMC) methods were also applied by (Özdemir et al.). Doping of injection layers reduces charge injection barriers and generates free charge carriers. Dopant concentration and host Fermi level were on the charge injection barrier and overall device conductivity. Particularly vital to this contribution is the perspective of optimizing the material system rather than individual components.

Nguyen et al. reviewed the integration between machine learning (ML) techniques and coarse-grained molecular dynamics (CGMD) simulations which is vital for developing polymer materials. These hybrid techniques reinforce the monomer sequence-functional behavior relationship of polymers and demonstrate the usage of these methods in development of new polymeric materials. This review covers three important topics in depth; polymeric configuration characterization, feed-forward property prediction, and inverse design with a focus on polymer systems and designs used in organic photovoltaics (OPVs).

The editors believe that the articles contained in this issue provide a number of important contributions in the design of organic optoelectronic materials. The increasing congruity between artificial intelligence/machine learning- and physics-based methods for generating design has been highlighted in every article in this Research Topic. We believe that the examples in these articles provide prototypes for utilizing these methods in new studies.

